# Dysglycemia, Dyslipidemia, and Pulmonary Tuberculosis Outcomes: Clinical Associations and Proposed Mechanisms

**DOI:** 10.3390/microorganisms14102408

**Published:** 2026-10-21

**Authors:** Zi-Jing Wu, Jian-Ming Wang, Xiao-Wei Deng

**Affiliations:** 1 Department of Epidemiology, School of Public Health, Nanjing Medical University, Nanjing 211166, China; syy_wuzijing@163.com (Z.-J.W.); jmwang@njmu.edu.cn (J.-M.W.); 2 The Fourth People’s Hospital of Nanning, Nanning 530023, China

**Keywords:** pulmonary tuberculosis, dysglycemia, dyslipidemia, treatment outcomes, immune dysfunction, host–pathogen interaction

## Abstract

Tuberculosis (TB) remains a leading global infectious disease burden. The rising global burden of dysglycemia and dyslipidemia—which geographically converges with TB in many low- and middle-income countries—poses substantial challenges to clinical management. Dysglycemia, encompassing prediabetes and diabetes mellitus, is a well-established risk factor for pulmonary tuberculosis (PTB) and adverse treatment outcomes; dyslipidemia frequently co-occurs with dysglycemia, yet its relationship with PTB outcomes is complex and varies by lipid phenotype, timing of measurement, and disease stage. Clinical evidence indicates that in the context of TB-DM, comorbid dyslipidemia is associated with more severe pulmonary pathology, heightened systemic inflammation, and increased risks of drug-induced liver injury (DILI) and mortality, although the overall evidence base remains associative. At the mechanistic level, chronic hyperglycemia and altered lipid metabolism may impair anti-tuberculosis immunity through complementary pathways—including macrophage effector dysfunction, T-cell exhaustion involving the advanced glycation end products (AGEs)–receptor for AGEs (RAGE) axis, and lipid-mediated modulation of inflammatory signaling—although translational evidence from preclinical models to human populations remains limited. This narrative review synthesizes current clinical and mechanistic evidence on the associations between dysglycemia, dyslipidemia, and PTB outcomes, identifies key methodological limitations in the existing literature, and outlines priorities for future research, including prospective longitudinal studies and integrated metabolic profiling, to advance evidence-based management of PTB patients with comorbid metabolic disorders.

## 1. Introduction

Tuberculosis (TB), caused by *Mycobacterium tuberculosis (Mtb)*, is a major global health threat that primarily affects the lungs [1,2]. In 2024, an estimated 10.7 million people developed TB worldwide, while 8.3 million were newly diagnosed and notified; of these notified cases, 84% were pulmonary and 16% extrapulmonary [3]. Pulmonary tuberculosis (PTB), the predominant form of the disease, is the primary focus of this review.

### 1.1. Definitions of Metabolic Exposures

In this review, dysglycemia is used as an umbrella term encompassing prediabetes (impaired fasting glucose 100–125 mg/dL or glycated hemoglobin A1c (HbA1c) 5.7–6.4%) and diabetes mellitus (DM) (fasting plasma glucose ≥ 126 mg/dL, HbA1c ≥ 6.5%, or 2-h plasma glucose ≥ 200 mg/dL on oral glucose tolerance test (OGTT)), following current diagnostic criteria [4]. Hyperglycemia denotes the pathophysiological state of elevated glucose and is not used interchangeably with dysglycemia; transient or stress-induced hyperglycemia may occur in acute infection but does not constitute a chronic metabolic exposure.

For dyslipidemia, no single threshold is applied uniformly across TB cohorts. For definitional clarity, we adopt the National Cholesterol Education Program Adult Treatment Panel III (NCEP ATP III) framework, in which dyslipidemia denotes the presence of one or more lipid phenotypes: hypertriglyceridemia (fasting triglycerides (TGs) ≥ 150 mg/dL [≥1.7 mmol/L]), low high-density lipoprotein cholesterol (HDL-C) (<40 mg/dL [<1.0 mmol/L] in men or <50 mg/dL [<1.3 mmol/L] in women), elevated low-density lipoprotein cholesterol (LDL-C) (≥130 mg/dL [≥3.4 mmol/L]), total cholesterol (TC) ≥ 200 mg/dL (≥5.2 mmol/L), or current use of lipid-lowering medication [5]. Because primary studies employ heterogeneous definitions and measurement timepoints, the present review consolidates evidence by specific lipid phenotype rather than by a single global “dyslipidemia” label.

### 1.2. Epidemiological and Immunological Rationale

The global burden of dysglycemia and dyslipidemia is substantial and rising. In 2024, the global DM prevalence among adults reached 11.1% (~589 million people), with >80% residing in low- and middle-income countries where the vast majority of new cases are projected by 2050 [6]. Dyslipidemia parallels this trend, with individual lipid phenotypes affecting 19–38% of the general population [7] and comorbid dyslipidemia present in 60–66% of DM patients [8]. Notably, this metabolic epidemic geographically converges with TB: six of the current top 10 countries by diabetes burden also appear on the World Health Organization (WHO) list of high-TB-burden countries [3,6], sustaining a long-recognized overlap [9].

Dysglycemia and dyslipidemia are closely interlinked, with insulin resistance as the shared core mechanism [10]. Experimental and clinical evidence indicates that dysglycemia and dyslipidemia independently compromise host anti-TB immunity: dyslipidemia impairs Mtb-specific T helper 1 (Th1) cytokine responses during latent tuberculosis infection (LTBI) [11], while comorbid uncontrolled DM further dysregulates macrophage effector functions in active PTB [12]. These immune defects translate into poorer clinical outcomes—TB–DM patients face elevated risks of death (odds ratio [OR] 1.88), recurrence (OR 1.64) [13], and multi-drug-resistant TB (MDR-TB) (OR 1.98) [13] compared with TB alone. Given this epidemiological and immunological convergence, a synthesis of the clinical associations between concurrent dysglycemia, dyslipidemia, and PTB outcomes is warranted.

### 1.3. Aims

This narrative review therefore aims to summarize the clinical associations between dysglycemia, dyslipidemia, and PTB outcomes, and to discuss proposed mechanisms underpinning these relationships, while identifying key knowledge gaps and priorities for future research.

### 1.4. Literature Search and Selection

This narrative review was informed by the literature identified through searches of PubMed, Embase, and Web of Science (inception to August 2026) using combinations of terms related to TB (e.g., “pulmonary tuberculosis”, “*Mycobacterium tuberculosis*”), glucose and lipid metabolism (e.g., “diabetes mellitus”, “dysglycemia”, “dyslipidemia”, “insulin resistance”), and clinical or mechanistic outcomes (e.g., “macrophage”, “T-cell immunity”, “comorbidity”); additional studies were identified through manual screening of reference lists; no formal systematic protocol, predefined eligibility criteria, or risk-of-bias assessment was performed.

## 2. Impact of Dysglycemia—With a Focus on Diabetes Mellitus—On PTB Clinical Outcomes and Underlying Mechanisms

Dysglycemia, particularly diabetes mellitus (DM), is a major host factor shaping both the occurrence and the clinical course of TB. Globally, an estimated 0.93 million incident TB cases in 2024 were attributable to diabetes—the second leading attributable risk factor after undernutrition [3]. The WHO has recognised diabetes as an important yet neglected risk factor for TB and, as one of the non-communicable disease determinants that must be addressed, regards it as a barrier to achieving the End TB Strategy target of a 90% reduction in TB incidence by 2035 [14], particularly in low- and middle-income countries, where the diabetes burden is growing most rapidly [6,14]. Against this background, this section synthesises recent original studies to delineate the impact of DM on clinical outcomes in PTB and the underlying mechanisms, while acknowledging that the dysglycemia spectrum extends beyond established DM.

### 2.1. Clinical Outcomes of PTB in the Context of Dysglycemia, with a Focus on Diabetes Mellitus

Dysglycemia encompasses DM and prediabetes; however, the clinical outcome evidence base is dominated by studies of established DM. This section therefore uses DM as the primary exemplar, noting where prediabetes data exist.

#### 2.1.1. Association of DM with Clinical Symptoms and Pulmonary Imaging Findings in PTB Patients

The influence of DM on clinical symptoms manifests primarily as a spectrum ranging from non-specific symptoms to exacerbated typical symptoms, a trajectory that, where the level of glycemic control has been examined, appears closely tied to it (Table 1). In patients with well-controlled glycemia, constitutional symptoms such as weight loss may persist even in the absence of more specific signs such as night sweats and lower-zone lesions [15]. Patients with DM and PTB frequently present with non-specific symptoms such as fatigue, weight loss, and mild anemia [16], while hyperglycemic status is significantly associated with a greater frequency of typical symptoms and higher symptom-based TB severity scores [17]. Glycemic indicators show positive associations with multiple markers of disease severity, with the most pronounced abnormalities observed in individuals with poor glycemic control [18].

The association between DM and imaging findings is particularly pronounced, as detailed in Table 1. Computer-aided chest radiograph analysis demonstrates higher TB abnormality scores in patients with diabetes [19], while cavity disease and non-upper-zone cavitation show borderline-significant trends in this population [19]. Patients with tuberculosis–diabetes mellitus (TB-DM) exhibit significantly more frequent cavitary disease [20] as well as more severe consolidation, bronchiectasis, and multilobar involvement [21]. Notably, these severe imaging abnormalities are frequently not paralleled by significant differences in clinical symptoms [21]. However, as summarized in Table 1, the available evidence is dominated by non-longitudinal designs; although most studies performed multivariable adjustment for demographic and lifestyle factors, diabetes duration, glucose-lowering regimens, and dyslipidemia were inconsistently or inadequately addressed across the included studies—variables that may independently influence pulmonary imaging manifestations. Therefore, clinicians should maintain a low threshold for chest imaging in TB-DM patients with suboptimal glycemic control, even when respiratory symptoms are atypical, although prospective studies are needed to establish the cost-effectiveness of such an approach.

#### 2.1.2. Association of DM with Treatment Outcomes in Patients with PTB

Accumulating evidence indicates that DM, particularly when poorly controlled, adversely affects treatment outcomes in adults with PTB, encompassing delayed bacterial clearance, elevated risks of treatment failure and post-treatment recurrence, and—in observational studies of MDR-TB—poorer prognoses. Conversely, optimal glycemic control appears to improve selected outcomes, although its protective role against recurrence and in MDR-TB remains to be validated (Table 2).

Bacterial clearance is consistently delayed in patients with DM across different monitoring modalities, including sputum culture and molecular bacterial load assays [22,23] (Table 2). Notably, this delay occurs despite a lower baseline bacterial burden in the DM group, suggesting impaired clearance kinetics rather than a higher initial bacillary load as the predominant mechanism [23]—a paradox that remains mechanistically incompletely understood. Suboptimal glycemic control (HbA1c ≥ 7%) amplifies these risks, whereas optimal glycemic control confers measurable benefits across multiple treatment outcomes, sputum positivity, and cavitary lesion resolution [24,25] (Table 2). It should be noted, however, that glycemic thresholds defining “poor control” varied across these studies, and few accounted for diabetes duration or concurrent glucose-lowering therapy, rendering direct comparisons across studies tenuous.

Regarding recurrence, DM has been consistently identified as an independent risk factor [26] (Table 2), although whether this risk is modified by glycemic control remains uncertain.

The picture is more nuanced in MDR-TB. Trial data indicate that comorbid DM substantially increases the burden of serious adverse events while efficacy outcomes remain comparable [27] (Table 2). In contrast, pooled observational analyses suggest that DM increases the risk of unfavorable outcomes in drug-resistant tuberculosis (DR-TB) and MDR-TB [28] (Table 2)—a discrepancy that likely reflects confounding by comorbidity burden in real-world cohorts rather than a true biological difference. Older patients appear particularly vulnerable, with worse prognostic profiles across multiple dimensions when DM coexists [29] (Table 2).

Nevertheless, these conclusions derive predominantly from observational studies with modest sample sizes and marked heterogeneity in glycemic assessment (HbA1c vs. fasting glucose), outcome definitions, and follow-up duration. The mechanistic link between delayed clearance and adverse outcomes remains largely inferential, and the apparent protective effect of optimal glycemic control may be partly attributable to confounding by health-seeking behavior and access to care. Causal inferences therefore warrant confirmation through high-quality prospective cohort studies with standardized glycemic monitoring and adequately powered trials in MDR-TB.

The adverse prognostic impact of chronic glucose dysregulation may extend below the diagnostic threshold for DM. Prediabetes has been associated with increased unfavorable treatment outcomes in a meta-analysis [30], although not consistently with mortality (Table 2). Evidence on glycemic trajectories during treatment is more tentative: transient hyperglycemia was associated with unfavorable outcomes in one prospective cohort, whereas persistent hyperglycemia was not [31], and diabetes treatment itself appeared strongly protective [31,32] (Table 2). Definitive assessment requires serial glycemic monitoring with standardized diabetes treatment protocols.

The above clinical evidence indicates that dysglycemia is associated with adverse PTB treatment outcomes across multiple clinical dimensions, with poor glycemic control and older patients being disproportionately affected. Nevertheless, the mechanisms by which chronic hyperglycemia impairs anti-TB immune responses remain incompletely elucidated—in particular, how sustained hyperglycemia modulates innate immunity (especially macrophage function) and *Mtb*-specific T-cell responses—and warrant further investigation.

### 2.2. Potential Mechanisms Underlying the Impact of Dysglycemia on PTB Clinical Outcomes

#### 2.2.1. T Cell Exhaustion as a Central Node in Diabetes-Associated PTB Susceptibility

Defects in adaptive immune responses, particularly T-cell dysfunction, are established contributors to persistent TB, treatment failure, and recurrence. Emerging evidence suggests that chronic hyperglycemia may exacerbate T-cell exhaustion, thereby compounding these risks [33,34]. The proposed mechanisms linking glucose dysregulation to impaired T-cell function are outlined below and chronic hyperglycemia—the primary pathological feature of DM—may compromise anti-TB immunity through multiple pathways. Specifically, hyperglycemia is proposed to promote T-cell memory exhaustion, thereby compromising the host’s long-term immune control of *Mtb*. This concept not only highlights the potential immunotoxicity of hyperglycemia but also provides a framework for understanding how a range of glucose metabolism disorders, including prediabetes and DM, may influence clinical outcomes in PTB.

#### 2.2.2. TCR-Dependent Signaling and the AGEs–RAGE Axis

Persistent *Mtb* antigen exposure activates two parallel T cell receptor (TCR)-dependent branches: one engaging nuclear factor of activated T cells (NFAT) signaling, and the other inducing programmed cell death protein-1 (PD-1) and inhibitory receptor expression. These branches cooperatively drive metabolic reprogramming in exhausted T cells, characterized by suppressed glycolysis and augmented fatty acid oxidation (FAO) in defined subsets [33]. Concurrently, chronic hyperglycemia activates the advanced glycation end products (AGEs)/receptor for advanced glycation end products (RAGE) axis primarily within antigen-presenting cells (APCs), triggering reactive oxygen species (ROS) production and nuclear factor kappa B (NF-κB) activation—a process highlighted within the TB-DM research agenda [34,35].

#### 2.2.3. TOX/PDCD1 Transcriptional Reprogramming and T Cell Subset Remodeling

Synthesizing evidence from chronic infection and oncology, we posit that persistent TCR signaling drives transcriptional reprogramming marked by thymocyte selection-associated high mobility group box protein (TOX) and programmed cell death 1 gene (PDCD1) upregulation [33], a process exacerbated by the AGEs–RAGE-derived pro-inflammatory milieu. This unified program is proposed to precipitate two interrelated sequelae:

1. Memory T cell exhaustion, defined by elevated PD-1 [36,37], impaired polyfunctionality (IFN-γ [interferon gamma], TNF-α [tumor necrosis factor alpha], IL-2 [interleukin-2]) [11,38], and dynamic C-X-C chemokine receptor type 5 (CXCR5) modulation restored upon preventive therapy [37];
2. T cell subset remodeling, characterized by naïve contraction and central memory T cell (Tcm) expansion [39].

**Figure 1:**
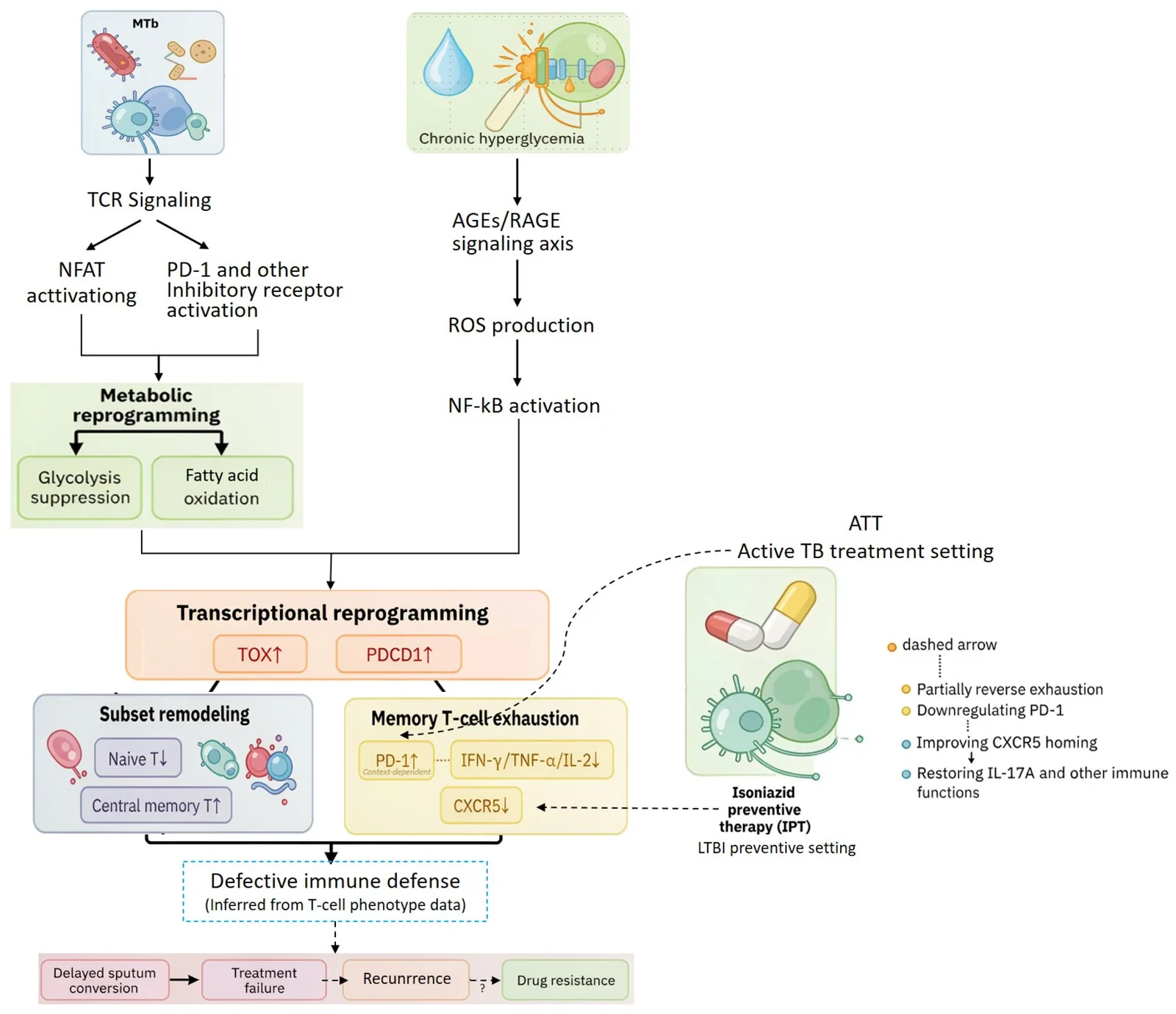
Proposed model: T cell dysfunction and therapeutic nodes in PTB–DM comorbidity. This figure presents a proposed conceptual model synthesizing current evidence on DM-related immunometabolic dysregulation in TB. Not all pathways represent definitively established causality; solid lines indicate relatively well-supported relationships, while dashed lines denote hypothesized or partially characterized linkages. The “Defective immune defense” node integrates observed T-cell defects and is labeled as partially inferred from T-cell phenotype studies. Persistent *Mtb* antigens engage the T cell receptor (TCR), activating nuclear factor of activated T cells (NFAT) signaling and programmed cell death protein-1 (PD-1)/inhibitory receptor expression, while hyperglycemia activates the advanced glycation end-products/receptor for AGEs (AGE–RAGE) axis in antigen-presenting cells (APCs). TCR signaling drives thymocyte selection-associated high mobility group box protein (TOX) and programmed cell death 1 gene (PDCD1) transcriptional programs and metabolic rewiring (glycolysis↓, fatty acid oxidation [FAO]↑), exacerbated by RAGE-derived inflammation. These processes converge into memory T-cell exhaustion (PD-1↑ [context-dependent], polyfunctionality↓, C-X-C chemokine receptor type 5 [CXCR5] dynamics) and subset remodeling (naïve↓, central memory T cells [Tcm]↑), which map to adverse clinical endpoints grouped as clinical outcomes (proposed linkage), including delayed sputum conversion, treatment failure, and recurrence. The direct relationship from recurrence to drug resistance is depicted as a dashed arrow, reflecting a hypothesized linkage with insufficient direct evidence. Dashed arrows denote interventions in two distinct clinical settings: (1) Active PTB treatment setting—ATT downregulates PD-1; (2) LTBI preventive setting (with DM)—isoniazid preventive therapy (IPT) restores CXCR5/interleukin-17A (IL-17A) and downregulates PD-1. Upward and downward arrows (↑/↓) indicate relative increase and decrease, respectively. Notably, PD-1 is annotated “(context-dependent)” in the figure; see Section 2.2.5 (PD-1 axis modulation) for discussion of context-dependent checkpoint modulation [42,43]. Abbreviations: ATT, anti-tuberculosis treatment; DM, diabetes mellitus; LTBI, latent tuberculosis infection.

Collectively, these perturbations may undermine antimycobacterial immunity and are associated with adverse outcomes, including delayed sputum conversion, treatment failure, and recurrence [40]. Critically, AGEs–RAGE-driven inflammation represents a putative driver of the immunometabolic dysregulation underlying these poor trajectories [34], as integrated into the proposed model (Figure 1).

#### 2.2.4. Reversibility of Exhaustion Phenotypes Under Standard Therapy

*Mtb*-induced memory T cell exhaustion may be partially reversible. In PTB, standard anti-tuberculosis treatment (ATT) downregulates PD-1 on CD4^+^ T cells [41]. In latent TB infection with DM (LTBI–DM), isoniazid preventive therapy (IPT) upregulates CXCR5 expression, restores interleukin-17A (IL-17A) production, and downregulates PD-1, normalizing immune parameters [37]. These data substantiate treatment-induced plasticity, indicating partial phenotypic reversal; however, definitive evidence for complete reversion remains lacking [37]. Notably, ATT and IPT operate in distinct clinical contexts (active PTB vs. LTBI), and while the proposed model integrates these therapeutic nodes, the settings are conceptually separate. Furthermore, while recurrent disease and drug resistance are clinically interrelated, the direct causal pathway from recurrence to drug resistance remains insufficiently supported; this relationship is depicted as a hypothesized (dashed) linkage in the proposed model (Figure 1).

#### 2.2.5. Context-Dependent PD-1 Checkpoint Dynamics and Safety Considerations

PD-1 axis modulation in TB is highly context-dependent. As detailed in Section 2.2.4, standard therapies in both active PTB (ATT) and LTBI–DM (IPT) dynamically modulate exhaustion markers including PD-1, CXCR5, and IL-17A [37,41]. These findings confirm that exhaustion phenotypes are plastic and context-dependent during standard treatment. However, direct checkpoint targeting carries safety risks: PD-1 blockade increased *Mtb* growth via excessive TNF-α in a human 3D granuloma model [42], whereas combining PD-1 and programmed death-ligand 1 (PD-L1) blockade with ATT restored polyfunctional CD4^+^ T cells and reduced bacillary burden in mouse models [43]. Thus, PD-1 signaling balances T-cell exhaustion and effector inflammation. Exhaustion-marker dynamics identify biologically relevant pathways but do not automatically define safe therapeutic targets; checkpoint-directed host-directed therapy requires careful evaluation of anti-mycobacterial efficacy versus immunopathology risk.

#### 2.2.6. Summary and Future Perspectives

In conclusion, the TCR-dependent exhaustion program and AGEs–RAGE axis do not operate in isolation; instead, they converge to compromise antimicrobial immunity. This framework, integrated as a proposed model (Figure 1), explains the heightened failure and recurrence risk in DM and provides a lens to understand glucose-driven immune remodeling. Current evidence remains largely cross-sectional, limiting causal inference; longitudinal studies integrating multi-omics and pharmacokinetic monitoring are needed to establish temporal relationships between dysglycemia, immune phenotypes, and treatment outcomes. Future efforts must identify predictive immune–metabolic signatures and evaluate host-directed therapies (HDTs) targeting the RAGE axis or selectively modulating (rather than uniformly reversing) T cell exhaustion, with particular emphasis on balancing anti-mycobacterial efficacy against immunopathology risk.

## 3. Dyslipidemia in PTB: Clinical Associations and Underlying Mechanisms

The epidemiological burden of hypolipidemia—particularly hypocholesterolemia—in PTB remains incompletely characterized. The paucity of large-scale, population-based surveys limits reliable prevalence estimates, as existing evidence derives predominantly from small case–control or cross-sectional studies with variable definitions and limited comparability. For instance, a single-center cross-sectional study in Uganda reported hypocholesterolemia (low TC) in 57.3% of treatment-naïve PTB patients [44]. In contrast, a nationwide Korean cohort study (*n* ≈ 5,000,000; mean follow-up 8.2 years) identified low TC as an independent predictor of incident PTB, with the lowest TC quartile demonstrating an adjusted hazard ratio (HR) of 1.35 (95% confidence interval [CI] 1.31–1.39) relative to the highest quartile [45]. Importantly, these two study types address fundamentally different questions: the Ugandan study describes a cross-sectional association during active disease, while the Korean study examines low TC as a pre-existing risk factor for developing TB. This inter-study heterogeneity likely arises from population stratification, variable definitions of dyslipidemia, differences in the timing of lipid measurement relative to TB diagnosis and treatment initiation, and divergent statistical methodologies.

Given the bidirectional interplay between host lipids and *Mtb*—coupled with the inherent limitations of observational designs—it remains unclear whether low TC represents a causal risk factor, a consequence of active disease (reverse causation), or both. Furthermore, whether hypolipidemia and specific lipid perturbations are independently associated with treatment response, sputum culture conversion, and recurrence risk remains uncertain. The extent to which such associations, if confirmed, are mediated by immunometabolic mechanisms—such as altered macrophage cholesterol homeostasis and impaired TCR signaling—requires investigation in human studies, as current evidence derives primarily from animal models and in vitro experiments.

Despite the availability of glycemic management guidance for PTB–DM—including the TB–DM comorbidity consensus [46] and guidelines from bodies such as the Chinese Diabetes Society—lipid management recommendations specifically tailored to PTB patients, both with and without DM, remain largely absent. Clarifying how hypolipidemia and specific lipid alterations relate to T cell and macrophage function and clinical outcomes would provide a theoretical foundation for developing evidence-based lipid management strategies in PTB. However, given the unresolved issue of reverse causation and the lack of interventional data, such strategies remain a long-term goal rather than an immediate clinical recommendation.

### 3.1. Association of Specific Lipid Phenotypes with PTB Clinical Outcomes

The association between specific lipid phenotypes and clinical outcomes of PTB has been examined primarily in cross-sectional studies, with comparatively fewer prospective cohort studies and interventional trials available to date.

#### 3.1.1. TC, HDL-C/LDL-C Perturbations, and Treatment Outcomes

TC and Radiographic Severity: In drug-resistant tuberculosis (DR-TB), serum TC levels were inversely correlated with radiographic severity scores, with lower TC associated with more extensive parenchymal involvement [47]. A similar pattern was observed in drug-sensitive PTB, where TC was inversely correlated with sputum smear positivity grade [48]. Both findings are derived from cross-sectional designs and cannot establish the temporal direction of the observed associations.

HDL-C and LDL-C Perturbations: HDL-C and LDL-C levels were also inversely correlated with radiographic severity in DR-TB [47], and both HDL-C and LDL-C were inversely correlated with the radiological extent of disease in drug-sensitive PTB [48]. In addition, low TC, HDL-C, and LDL-C levels paralleled greater inflammatory burden in patients with active PTB [49]. Collectively, these cross-sectional data indicate that hypolipidemia parallels greater radiographic disease extent and higher semi-quantitative sputum bacillary burden, and elevated inflammatory markers, but causality cannot be inferred.

Treatment Failure, Mortality, and Lipid-Based Interventions: Regarding treatment outcomes, a small North Indian cohort comparing PTB patients with and without dyslipidemia reported higher rates of treatment failure and mortality in the dyslipidemia group [50]. However, these crude comparisons lacked adjustment for baseline confounders and should be interpreted descriptively rather than causally. A larger retrospective cohort from Taiwan (NTUH, National Taiwan University Hospital) reported that higher baseline TC and HDL-C tertiles were independently associated with lower all-cause and infection-related mortality after multivariable adjustment, along with reductions in inflammatory markers [51] (see Table 3 for detailed effect estimates). As an observational study, residual confounding cannot be excluded. A pilot randomized controlled trial suggested that supplementing standard anti-TB treatment with a cholesterol-enriched diet accelerated sputum culture conversion [52]. However, this trial was small and single-center; confirmation in adequately powered, multi-center trials is needed before clinical translation.

Limitations and Causal Inference: Crucially, the interplay between hypolipidemia and inflammation in PTB must be interpreted with caution. While low TC, HDL-C, and LDL-C are consistently observed in cross-sectional studies [48,49], causality cannot be inferred from these designs alone. Active PTB is a catabolic, inflammatory disease that can itself profoundly alter host lipid metabolism. Therefore, the notion that hypolipidemia directly drives inflammation risks conflating cause with consequence: low cholesterol may be a result of infection-induced wasting and hepatic dysfunction rather than a causal contributor to poor outcomes. Disentangling these possibilities requires prospective studies with serial lipid measurements from before TB diagnosis through treatment completion.

#### 3.1.2. Lipidomic Profiles and Treatment Failure

Beyond global dyslipidemia, specific lipid species may represent distinct phenotypic markers. A prospective nested case–control study using untargeted lipidomics reported that patients who experienced treatment failure had lower cholesteryl esters (CEs) and oxylipins, alongside elevated ceramides and select TG species; a two-lipid CE classifier achieved a moderate area under the curve (AUC) for predicting treatment failure [53] (Table 3). It is important to note that this classifier identifies a correlative signature rather than a causal mechanism; the predictive performance requires external validation in independent, geographically diverse cohorts before clinical application.

In summary, hypolipidemia and specific lipid perturbations are associated with clinical severity and poor prognosis in PTB across multiple cross-sectional and cohort studies. Low TC and HDL-C levels have been independently associated with increased risks of treatment failure and mortality in adjusted analyses, although residual confounding cannot be excluded. Comprehensive lipidomics offers a promising research tool for risk stratification, but routine clinical monitoring of standard lipid panels should be regarded as a candidate for prospective evaluation rather than an established clinical practice. Key prerequisites—including validated actionable thresholds, optimal monitoring intervals, incremental predictive value beyond existing clinical markers, and evidence that acting upon lipid results improves patient outcomes—remain to be determined. A critical distinction must be made between acute, infection-driven consumptive hypolipidemia and pre-existing metabolic dyslipidemia, as these entities may require divergent host-directed therapeutic approaches. At present, lipid monitoring in PTB should be reserved for research contexts until prospective evidence demonstrates clinical utility.

### 3.2. Potential Mechanisms Linking Hypolipidemia and Specific Lipid Alterations to PTB Clinical Outcomes

Most mechanistic studies of lipid alterations in the absence of concurrent DM on PTB outcomes derive from in vitro experiments and animal models and lack large-scale population evidence. Some mechanisms—e.g., lipid droplet and foam cell formation—are triggered by *Mtb* infection, but elevated circulating lipids provide substrates that may worsen them, thus representing indirect effects of dyslipidemia. Lipid alterations may affect anti-TB immunity through the following pathways. Notably, the mechanistic pathways discussed below are derived predominantly from in vitro and animal models and should be considered speculative hypotheses rather than established causal mechanisms in human PTB.

#### 3.2.1. Macrophage Function and Cholesterol Homeostasis

Plasma-membrane cholesterol is required for *Mtb* uptake by macrophages [54]; following entry, coronin-1 (TACO) is recruited to the mycobacterial phagosome, where its signalling sustains phagosomal arrest [55]. In subsequent work using coronin-1-deficient macrophages and calcineurin inhibitors, Coronin-1 was shown to activate Ca^2+^-dependent calcineurin signalling, thereby blocking phagosome–lysosome fusion and enabling bacterial persistence [55]. Independently, *Mtb* infection or exposure to mycobacterial lipids disrupts intracellular cholesterol trafficking in macrophages, causing cholesterol accumulation in lysosomes; this sequesters major histocompatibility complex (MHC) class II in lysosomes, impairs antigen presentation, and dampens anti-*Mtb* CD4^+^ T cell activation [56].

Thus, at the macrophage level, cholesterol homeostasis links two distinct axes of immune evasion: an innate axis in which Coronin-1/calcineurin signalling arrests phagosomal maturation [55] (with membrane cholesterol required for initial bacterial uptake [54]), and an adaptive axis in which infection-driven cholesterol accumulation traps MHC-II and weakens T cell help [56]. Notably, a circulating lipid profile does not directly measure lysosomal cholesterol accumulation within macrophages, nor does it prove that systemic dyslipidemia caused the observed cellular defect. Circulating lipids serve primarily as upstream substrates; intracellular cholesterol handling reflects infection-driven trafficking perturbations that cannot be inferred from host systemic lipid concentrations alone. Importantly, active PTB is a catabolic inflammatory disease that can itself profoundly alter host lipid metabolism—a reverse-causality consideration that must be distinguished from pre-existing metabolic dyslipidemia when interpreting observational lipid–outcome associations.

#### 3.2.2. Foam Cell Formation and Lipid Droplet Functions in Mycobacterial Persistence

Foam macrophage formation in tuberculous granulomas is actively driven by *Mtb* infection. Oxygenated mycolic acids (OMAs) in the bacterial cell wall directly trigger macrophage conversion into lipid droplet-rich foam cells, which constitute a nutrient-rich reservoir for bacterial persistence [57]. These lipid droplets act not only as storage depots for TG and cholesterol but can also be induced via the Toll-like receptor 2 (TLR2)–peroxisome proliferator-activated receptor γ (PPARγ) pathway [58]. Functionally, lipid droplets serve as platforms for prostaglandin E_2_ (PGE_2_) synthesis, which suppresses Th1 immune responses [58]. Rab7 (Ras-related protein Rab-7a) and its effector RILP (Rab-interacting lysosomal protein) are recruited to lipid droplets, where they mediate lipid droplet–phagosome interactions that promote mycobacterial survival [58]. This local, infection-driven lipid accumulation is distinct from host systemic dyslipidemia. However, in individuals with DM, altered systemic metabolism—including, but not limited to, circulating lipid alterations—may provide additional exogenous substrates that augment the intra-granulomatous lipid pool, thereby promoting caseous necrosis and bacterial persistence. While the clinical association between DM and severe TB outcomes is well established [16,59], the specific contribution of circulating lipids versus other DM-related metabolic perturbations (e.g., hyperglycemia, insulin resistance) to granuloma lipid accumulation has not been established and requires dedicated experimental investigation.

#### 3.2.3. Ceramide and Nucleotide-Binding Oligomerization Domain-like Receptor Family, Pyrin Domain-Containing 3 (NLRP3) Inflammasome Activation

Ceramide, an intermediate of lipid metabolism, activates the NLRP3 inflammasome in macrophages via the acid sphingomyelinase (ASM)–ceramide (Cer)–thioredoxin-interacting protein (TXNIP) signaling axis, driving interleukin-1β (IL-1β) and interleukin-18 (IL-18) secretion [60]. This pathway has been validated in lipopolysaccharide (LPS)/ adenosine triphosphate (ATP)-stimulated macrophage models [60]; however, whether and how this axis operates during *Mtb* infection remains to be elucidated.

#### 3.2.4. Lipid Alterations Exert Dual Effects on T Cell Responses in TB

Lipid alterations also directly impact T cell responses. Among conventional T cells, T helper 17 (Th17) cell differentiation is particularly sensitive to the lipid microenvironment: a high-fat diet induces acetyl-CoA carboxylase 1 (ACC1)-dependent reprogramming of fatty acid synthesis, which modulates retinoic acid receptor-related orphan receptor γt (RORγt) function and thereby promotes Th17 differentiation. A positive correlation between interleukin-17A (IL-17A)-producing CD45RO^+^ memory CD4^+^ T cells and acetyl-CoA carboxylase alpha (ACACA; encoding ACC1) expression was observed in individuals with obesity [61].

This “double-edged sword” effect extends beyond conventional T cells. A 2025 study using a humanized double-transgenic model expressing group-1 CD1 and a mycolic acid (MA)-specific, CD1b-restricted double-negative 1 T cell receptor transgenic (DN1Tg) demonstrated that diet-induced lipid perturbations enhance TCR signaling and glycolysis, leading to enhanced IFN-γ production by MA-specific CD1b-restricted T cells and improved mycobacterial control in vitro. However, dyslipidemia concurrently promotes apoptosis of these T cells, resulting in a marked reduction in DN1Tg T cell numbers and increased pulmonary bacterial burden in an in vivo *Mtb* infection model, ultimately impairing lipid antigen-specific T cell-mediated protective immunity [62].

Most of these findings derive from in vitro or animal models. Whether they translate to PTB patients with dyslipidemia in the absence of diabetes warrants prospective cohort studies complemented by mechanistic investigations. Future animal models should recapitulate clinical hypocholesterolemia or specific lipid profiles to identify causal targets.

## 4. Impact of Comorbid Metabolic Disorders on PTB Clinical Outcomes

### 4.1. Impact of Concomitant Dysglycemia and Dyslipidemia on PTB Clinical Outcomes: A Focus on TB-DM

Concomitant dysglycemia and dyslipidemia remain a neglected yet critical aspect of TB-DM management. Dyslipidemia accompanies 60–90% of DM cases (up to 88.9% in type 2 diabetes mellitus [T2DM]) [63], and DM raises PTB incidence 1.5–2.4-fold [59]; once PTB is established, coexisting DM increases death risk approximately 1.9-fold (adjusted odds ratio [aOR] 1.88, 95% CI 1.59–2.21) [13]. However, how concurrent glucose and lipid abnormalities jointly shape PTB treatment outcomes remains poorly understood. The evidence is summarized below.

The evidence below is organized by the specific lipid phenotype implicated, while acknowledging that composite indices (e.g., the triglyceride–glucose [TyG] index) simultaneously reflect both metabolic domains. Detailed study characteristics and effect estimates are provided in Table 4 (Parts 1–2).

#### 4.1.1. Hypertriglyceridemia and the TyG Index

Converging cross-sectional and cohort data indicate that PTB patients with coexisting dysglycemia and dyslipidemia exhibit more extensive pulmonary involvement and heightened systemic inflammation than those with either metabolic disturbance in isolation. Composite indices that jointly capture glucose–lipid perturbations—particularly the TyG index—are consistently associated with higher sputum smear grades, larger cavitary lesions, and advanced radiographic scores at diagnosis [64]. In T2DM-PTB cohorts, the highest TyG quartile conferred markedly elevated odds of multiple and thick-walled cavities compared with the lowest quartile [64], while prediabetes and diabetes were both linked to greater radiographic lesion diversity [65]. These findings are summarized in Table 4 (Part 1).

#### 4.1.2. Pro-Atherogenic Profiles (VLDL, ApoB)

The pro-atherogenic lipid profile characteristic of TB–DM comorbidity—including elevated very low-density lipoprotein (VLDL) and apolipoprotein B (ApoB)—persists beyond the acute treatment phase and correlates with radiographic severity [66] (Table 4, Part 2).

#### 4.1.3. HDL-C and LDL-C Perturbations

Low HDL-C and low LDL-C further compound pulmonary pathology. In case–control analyses, low LDL-C was associated with enlarged caseous necrosis, while low HDL-C showed a trend toward severe perinecrotic fibro-encapsulation [67] (Table 4, Part 2).

#### 4.1.4. Caveats

While these observations suggest that combined glucose–lipid abnormalities exert adverse effects on pulmonary pathology, the predominance of observational designs precludes causal inference, and the overall evidence base remains associative. Prospective longitudinal data directly linking baseline glucose–lipid profiles to subsequent radiographic progression are scarce, leaving unresolved whether these associations denote a direct pathogenic mechanism or merely reflect a more advanced disease phenotype at presentation.

### 4.2. Other Adverse Outcomes: Drug-Induced Liver Injury and Cardiovascular Risk

Beyond pulmonary manifestations, metabolic disorders are associated with increased risk of extrapulmonary adverse outcomes during PTB treatment, particularly drug-induced liver injury (DILI) and sustained cardiovascular vulnerability. In prospective cohorts, individuals with metabolic abnormalities had a nearly threefold higher risk of DILI compared with metabolically healthy individuals [68]. Pro-atherogenic lipid profiles in TB–DM not only correlate with radiographic severity (Section 4.1.1) but also persist during treatment, implicating sustained cardiovascular vulnerability [66]. Conversely, higher baseline HDL-C and total cholesterol have been associated with lower all-cause and infection-related mortality in drug-sensitive PTB (DS-PTB) cohorts [51], suggesting a potential survival advantage of preserved lipid reserves in active PTB. Nevertheless, this evidence should be interpreted cautiously: reverse causation cannot be excluded, as active TB itself induces catabolic consumption that lowers lipid levels, and many studies fail to account for potential confounding by statin use or nutritional status. Full effect estimates are provided in Table 4 (Part 3), Table 5 and Table 6.

### 4.3. Potential Benefits of Metformin and Statin Use in PTB-DM Patients

Pharmacological interventions targeting glucose–lipid pathways have been explored as potential adjunctive strategies in PTB-DM management. Metformin use has been associated with reduced mortality in observational studies [66,70]; similarly, statin use has been associated with improved outcomes, particularly when combined with good glycemic control [71]. However, as non-randomized observations, these associations may be influenced by residual confounding including differences in healthcare access, comorbidity burden, and treatment selection; both require prospective validation. Mendelian randomization evidence further distinguishes causal determinants of PTB incidence from modifiable drivers of disease progression [69] (Table 5 and Table 7), highlighting the need to differentiate primary prevention from adjunctive therapeutic strategies. Bridging this incidence–outcome divide is essential for translating glucose–lipid modulation into effective TB control measures.

### 4.4. Methodological Limitations and Future Directions

Current evidence regarding concomitant dysglycemia and dyslipidemia in PTB is constrained by three methodological limitations that collectively compromise causal inference. First, cross-sectional designs predominate, precluding the establishment of temporal relationships and leaving unresolved whether reduced lipid levels represent a cause or a consequence of infection-driven catabolic consumption (i.e., reverse causation). Second, inconsistent definitions of dyslipidemia—ranging from arbitrary thresholds to composite indices—impede cross-study comparisons and obscure the independent contributions of specific lipid fractions. Third, uncontrolled confounding remains widespread: statin use, dietary intake, glycemic control, and nutritional status are seldom modeled jointly, such that observed lipid–outcome associations may reflect broader metabolic or pharmacological confounding rather than an independent effect of dyslipidemia.

These methodological limitations directly account for the persistent uncertainty regarding the independent versus interactive contributions of glucose and lipid abnormalities to *Mtb* susceptibility and treatment outcomes. This uncertainty is compounded by the frequent coexistence of dysglycemia (manifesting as chronic hyperglycemia) and heterogeneous dyslipidemia phenotypes, as well as the scarcity of experimental models capable of disentangling these disturbances. Whether concurrent dysglycemia and dyslipidemia interact at the immunological or clinical level remains unexamined; this gap is even more pronounced in non-diabetic populations, where the independent effect of specific lipid phenotypes on TB outcomes is largely unexplored. As discussed in a recent review (Ngo et al. [72]), dysglycemia and dyslipidemia closely interrelate in TB–DM comorbidity, but primary evidence distinguishing their independent versus combined effects is lacking. Future longitudinal cohorts should prioritize integrated metabolic profiling—distinguishing circulating lipid fractions, intracellular mycobacterial lipid accumulation, and acute illness versus pre-existing chronic metabolic states—to disentangle these pathways and determine whether combined metabolic modulation yields benefits beyond single-pathway targeting.

## 5. Conclusions and Perspectives

Dysglycemia (including DM and prediabetes) and dyslipidemia are major host-related risk factors that adversely affect the onset, progression, and treatment outcomes of PTB. Dysglycemia primarily drives adverse clinical outcomes through immune-mediated mechanisms, whereas dyslipidemia is likewise associated with poor prognosis. When these two metabolic disorders co-occur, they cluster within a broader metabolic–inflammatory phenotype that is associated with worsened PTB outcomes and long-term cardiovascular prognosis; whether this reflects interaction or mere coincidence of independent risk factors remains undefined.

Current evidence does not permit mechanistic conclusions regarding whether concurrent dysglycemia and dyslipidemia exert additive or synergistic effects on anti-tuberculosis immunity. The frequent clinical coexistence of these disturbances, combined with the methodological limitations outlined in Section 4.1.4, leaves unresolved whether integrated intervention strategies targeting both pathways simultaneously confer benefits beyond single-pathway approaches. Addressing this gap requires prospective studies designed a priori to disentangle glucose–lipid interactions at both the clinical and immunological levels. Notably, the clinical implications of dyslipidemia in PTB are context-dependent: pre-existing atherogenic profiles (e.g., elevated VLDL, ApoB) may represent legitimate therapeutic targets, whereas low circulating cholesterol during active infection may reflect an adaptive host response rather than a modifiable risk factor. Metabolic assessment and future interventions should therefore be phenotype-specific, distinguishing patients who may benefit from lipid modulation from those in whom indiscriminate lipid lowering could be detrimental.

Future research should prioritize several directions. First, early screening and integrated clinical management of comorbid dysglycemia and dyslipidemia in PTB patients must be strengthened. Second, basic and translational studies are needed to elucidate the molecular mechanisms by which hyperglycemia and dyslipidemia independently and jointly influence anti-tuberculosis immune defense. Third, prospective longitudinal cohort studies should be conducted to confirm the independent and combined effects of these metabolic disorders on PTB outcomes. Fourth, randomized controlled trials (RCTs) are warranted to evaluate the clinical efficacy and safety of combined metformin and statin therapy in this population (informed by prior observational studies [69,70]). Such trials must distinguish between established metabolic indications (e.g., glycemic control per diabetes guidelines, statin therapy per cardiovascular risk) and experimental, TB-directed metabolic benefits, ensuring that interventions targeting PTB outcomes are not conflated with routine metabolic management. In addition, HDTs and multi-omics integrative analyses may offer novel insights for precision medicine in comorbid PTB. Trial designs should additionally incorporate safety monitoring and assessment of potential drug–drug interactions between anti-tuberculosis agents and glucose-lowering or lipid-modifying medications. Ultimately, high-quality evidence is essential to drive clinical translation and improve the prognosis of PTB patients with concomitant dysglycemia and dyslipidemia.

## Figures and Tables

**Table 1:** Summary of studies on the association between diabetes mellitus and clinical symptoms or pulmonary imaging findings in patients with PTB.

Study (Reference)	Country	Study Design	Sample Size	Imaging Modality & Reader	Key Findings
Saalai et al., 2021 [15]	India	Cross-sectional; 6-month period	50 PTB + DM (5 good/45 poor glycemic control)	CXR; pulmonologists	Poorer glycemic control associated with larger/more cavities, lower-zone lesions (none in good control), and higher sputum smear grade; nonhomogenous opacity most common finding.
Tong et al., 2021 [16]	China	Multicenter cross-sectional (observational), 2015–2018	1417 newly diagnosed PTB (312 T2DM, 1105 non-DM)	Thoracic CT; reviewed by 2 radiologists and clinical specialists	TB-DM had higher TB severity score ≥ 3, more central lobular nodules, cavities (especially thick-wall), and tracheal-wall thickening; drug susceptibility testing profiles comparable. Elevated uric acid was independent risk factor for thick-wall cavity (OR 2.81, 95% CI 1.24–6.40).
Xu et al., 2024 [17]	China (Weifang, Shandong)	Cohort study, 2019–2022	791 PTB (266 hyperglycemia, 33.6%)	Chest CT by 2 radiologists and specialists; sputum AFB smear	Hyperglycemia associated with elevated TB score, cough, night sweats, and positive sputum smear (adjusted OR 1.33–1.94); CT lesion extent not affected; higher risk of vomiting, abdominal distension, constipation.
Meng et al., 2023 [18]	China (Chengdu)	Retrospective cohort, 2019–2021	3393 TB + DM (537 good/542 fair/2314 poor control); cavity 16.8%, sputum-positive 52.2%	Chest X-ray and CT; reader number/blinding not reported	Poor glycemic control linked to higher cavity rate (19.0% in poor group, 440/2314), higher sputum positivity (55.4%), and more intrapulmonary/both lesions; glycemia and TB severity influenced each other bidirectionally.
Geric et al., 2023 [19]	Pakistan (Karachi)	Secondary analysis of prospective TB diagnostic study, 2017–2018	272 culture-confirmed PTB (63 DM, 209 non-DM)	Digital CXR interpreted by CAD (qXR v3.0); culture reference standard	Computer-aided diagnosis CXR showed diabetes linked to higher TB abnormality score and more cavitary disease, particularly non-upper-zone cavities; other radiographic features comparable.
Wang et al., 2025 [20]	China (Zhejiang)	Multi-center observational, 2017–2019)	8421 newly diagnosed PTB (763 DM, 1833 pleuritis, 160 silicosis)	Chest X-ray; reader number/blinding not reported	DM associated with cavities (AOR 2.88, 95% CI 2.42–3.43) and M. tuberculosis-positive results (AOR 2.05, 95% CI 1.72–2.45); DM had highest cavity rate (54.8%).
Yang et al., 2023 [21]	China (Sichuan, Liangshan)	Retrospective cross-sectional	154 TB-DM matched to 145 TB-NDM (age ±3 y, sex)	Chest CT; 3 group-blinded radiologists	TB-DM had more severe CT disease (89.61% vs. 68.97%): consolidation, cavitary/thick-wall lesions, bronchiectasis, exudative and fibrous lesions more frequent; clinical symptoms and lobe distribution comparable. Conversely, enlarged/calcified hilar-mediastinal lymph nodes more common in TB-NDM.

Abbreviations: PTB, pulmonary tuberculosis; T2DM, type 2 diabetes mellitus; TB-DM, tuberculosis with comorbid diabetes mellitus; TB-NDM, tuberculosis without comorbid diabetes mellitus; AOR, adjusted odds ratio; OR, odds ratio; CI, confidence interval; CT, computed tomography; CXR, chest X-ray; CAD, computer-aided diagnosis; AFB, acid-fast bacilli; DST, drug susceptibility testing. Notes: good glycemic control defined as HbA1c ≤ 7%; poor control as HbA1c > 7% or fasting glucose above study-specific thresholds. Yang et al. [21] employed age (±3 years) and sex matching between TB-DM and TB-NDM groups; all other studies used consecutive or retrospective sampling.

**Table 2:** Summary of studies on the association of diabetes mellitus with treatment outcomes in patients with PTB.

Category	Key Finding	Primary Result	Definition/Criteria	Reference
Bacterial clearance	Delayed sputum culture conversion	60.0 vs. 52.0 days (*p* < 0.001); aHR 0.721 (95% CI 0.528–0.986), *p* = 0.040	First of two consecutive negative sputum samples (smear or culture) after treatment start	[22]
	Delayed TB-MBLA negativity	62 vs. 57 days (*p* = 0.022); day 56 load 1.41 vs. 0.73 log_e_CFU/mL (*p* = 0.028)	TB-MBLA negative: Cq > 30 (<1.0 log_e_CFU/mL)	[23]
	Poor glycemic control ↑ 3-month sputum positivity (delayed clearance)	RR 2.97 (95% CI 1.10–8.07), *p* = 0.03 (low certainty)	Sputum smear positivity at month 3	[24]
Treatment failure	Poor glycemic control ↑ treatment failure	RR 1.91 (95% CI 1.81–3.07), *p* = 0.008 (low certainty)	Failure: WHO-defined (smear/culture positive ≥ mo 5 or at end); exposure: HbA1c ≥7% vs. <7%	[24]
Glycemic control (protective)	Optimal glycemic control improved multiple TB outcomes	Improved outcomes RR 1.13 (95% CI 1.02–1.25); sputum positivity RR 0.23 (95% CI 0.09–0.61); cavitary lesions RR 0.59 (95% CI 0.51–0.68)	OGC defined as HbA1c <7% in most studies; one study used FPG < 7.0 mmol/L	[25]
Recurrence	DM as independent risk factor for TB recurrence	20.21 vs. 6.28 per 1000 PYs; aHR 2.40 (95% CI 1.68–3.45), *p* < 0.001	Active TB occurring ≥6 mo after successful initial treatment (cure/completion); recurrence not distinguished from reinfection	[26]
MDR-TB	↑ SAEs; efficacy comparable	SAE 41% vs. 22% (*p* < 0.001); FoR 8.3% vs. 5.9% (*p* = 0.38); aHR for FoR 1.38 (95% CI 0.51–3.73), *p* = 0.53	DAIDS (AE/SAE grading); STREAM Definite/Probable FoR; DM: self-report/RBG ≥ 200 mg/dL/DM meds	[27]
DR-/MDR-TB	DM ↑ unsuccessful outcomes	DR-TB OR 1.56 (95% CI 1.24–1.96); MDR-TB OR 1.57 (95% CI 1.20–2.04)	WHO outcomes: cured and completed defined as successful	[28]
Elderly PTB (>60 y)	Worse prognosis in elderly with PTB-DM	Sputum culture positivity 81.05% vs. 62.67% (*p* = 0.001); treatment duration 298 vs. 223 days (*p* < 0.001); cure rate 64.21% vs. 75.56% (*p* = 0.039); cavity rate 31.58% vs. 15.11% (*p* = 0.001)	WHO outcomes: cured and completed defined as success; age > 60 y; DM: T2DM	[29]
Prediabetes	Prediabetes ↑ unfavourable treatment outcomes	RR 1.41 (95% CI 1.02–1.96; I^2^ = 56%); all-cause mortality RR 1.59 (95% CI 0.75–3.38)	Meta-analysis of 8 prospective cohorts (*n* = 3001, 752 prediabetes); prediabetes per ADA criteria	[30]
Glycemic trajectory	Transient hyperglycemia ↑ unfavourable outcomes	Transient aIRR 2.07 (95% CI 1.04–4.15); persistent aIRR 1.64 (95% CI 0.71–3.79); DM treatment aIRR 0.38 (95% CI 0.15–0.95)	Prospective cohort, Pune, India; transient *n* = 26	[31]
Glycemic control (DM effect)	DM ↑ early mortality but not composite unfavourable outcome	DM aHR 4.36 (95% CI 1.62–11.76) for early mortality; aRR 1.13 (95% CI 0.75–1.70) for composite unfavourable outcome	Same Pune cohort; adjusted for confounders	[32]

Abbreviations: ↑, increased; PTB, pulmonary tuberculosis; DM, diabetes mellitus; aHR, adjusted hazard ratio; HR, hazard ratio; OR, odds ratio; RR, risk ratio; aIRR, adjusted incidence rate ratio; aRR, adjusted relative risk; CI, confidence interval; SAE, serious adverse event; FoR, failure or recurrence; DR-TB, drug-resistant tuberculosis; MDR-TB, multidrug-resistant tuberculosis; FPG, fasting plasma glucose; OGC, optimal glycemic control; DAIDS, Division of AIDS, National Institute of Allergy and Infectious Diseases; TB-MBLA, TB molecular bacterial load assay; Cq, quantification cycle; eCFU, equivalent colony-forming units. Notes: Reference groups: Diabetes vs. no diabetes comparisons (reference: non-DM patients): studies [22,23,26,27,28,29]. Glycemic-control-level comparisons (reference: controlled glycemia, HbA1c < 7% or equivalent): study [24] (bacterial clearance and treatment failure rows) and [25]. Prediabetes analysis: study [30]. Glycemic trajectory and DM prognosis (same cohort, Pune, India): studies [31,32].

**Table 3:** Summary of studies on lipid phenotypes and clinical outcomes in PTB.

Study (Reference)	Exposure Definition	Population	Timing	TB Stage	Outcome	Effect Estimate (Direction)	Confounders
Uganda single-center [44] (Uganda, 2018)	Hypocholesterolemia (low TC)	PTB patients, single-center	Baseline (treatment-naïve)	Active PTB	Prevalence of hypocholesterolemia (57.3%)	No effect estimate reported; descriptive only	Not reported
Korean nationwide cohort [45] (South Korea, 2021)	Low TC (lowest vs. highest quartile)	~5,000,000 general population; mean follow-up 8.2 y	Baseline (pre-TB)	Incident TB (mixed PTB and EPTB)	Incident TB	aHR 1.35 (1.31–1.39), *p* < 0.001	Adjusted (multivariable)
DR-PTB cross-sectional [47] (India, 2019)	TC, HDL-C, LDL-C (continuous)	DR-PTB patients, *n* = 33	Cross-sectional	Active DR-PTB	Radiological severity score (CXR score)	Inverse correlation: TC r = −0.546, *p* = 0.001; HDL-C r = −0.479, *p* = 0.005; LDL-C r = −0.431, *p* = 0.012	Not reported
PTB cross-sectional [48] (Turkey, 2007)	TC, HDL-C, LDL-C (continuous)	PTB patients vs. healthy controls; drug susceptibility not reported	Cross-sectional	Active PTB	Radiological extent (DRED); sputum smear positivity (DSP)	DRED: TC r = −0.43, HDL-C r = −0.60, LDL-C r = −0.28 (all *p* < 0.05). DSP: TC r = −0.36, HDL-C r = −0.43, LDL-C r = −0.32 (all *p* < 0.05)	Not reported
Inflammatory correlation study [49] (India, 2023)	Low TC, HDL-C, LDL-C	PTB patients	Cross-sectional	Active PTB	Inflammatory markers	Inverse correlation; direction only, no magnitude reported; causality uncertain	Not reported
North India DS-TB cohort [50] (India, 2024)	Dyslipidemia (clinical diagnosis)	72 with vs. 72 without dyslipidemia; DS-TB (PTB-predominant, EPTB included)	Baseline	Active DS-TB	Unfavorable outcome (composite)	RR 1.11 for unfavorable outcome	None (unadjusted)
NTUH retrospective cohort [51] (Taiwan, 2021)	TC, HDL-C (tertiles)	DS-PTB, *n* = 514; median follow-up 9 mo	Baseline	Active DS-PTB	All-cause mortality; infection-related mortality	Highest tertile protective: aHR 0.30 (TC) and 0.17 (HDL-C) for all-cause mortality; aHR 0.26 (TC) and 0.13 (HDL-C) for infection-related mortality	Adjusted (multivariable: age, sex, BMI, CCI, smoking, alcohol)
Cholesterol-enriched diet intervention [52] (Mexico, 2005)	Hypocholesterolemia (intervention: 800 mg/day cholesterol diet + ATT)	Newly diagnosed PTB	During treatment; endpoint at week 2	Active PTB	Sputum culture conversion	80% vs. 9% culture-negative by week 2; median 14 vs. 28 days	Randomization
Lipidomic nested case–control [53] (India, 2022)	32 baseline lipid species (CE, ceramides, oxylipins, TG)	192 DS-PTB patients	Baseline	Active DS-PTB	Treatment failure	Lower CE and oxylipins, higher ceramides and TG in failures; CE(16:0)+CE(18:2) classifier test-set AUC 0.79 (0.65–0.93)	Matched design

Abbreviations: aHR, adjusted hazard ratio; ATT, anti-tuberculosis treatment; AUC, area under the curve; BMI, body mass index; CCI, Charlson comorbidity index; CE, cholesteryl ester; CI, confidence interval; CRP, C-reactive protein; CXR, chest X-ray; DRED, radiological extent of disease score (originally validated in PTB [48]); DS-TB, drug-susceptible tuberculosis; DS-PTB, drug-susceptible pulmonary tuberculosis; DR-PTB, drug-resistant pulmonary tuberculosis; EPTB, extrapulmonary tuberculosis; HDL-C, high-density lipoprotein cholesterol; HR, hazard ratio; LDL-C, low-density lipoprotein cholesterol; PTB, pulmonary tuberculosis; TG, triacylglycerol; TC, total cholesterol. Notes: 1. Populations, lipid measures, and outcomes differ across studies and must not be extrapolated interchangeably: incident TB [45], DR-PTB [47], PTB without systematic drug-susceptibility testing [48], and DS-TB/DS-PTB [50,51,53]. Lipid assessments include routine clinical panels [45,47,48,49,50,51] and unbiased lipidomics [53]; the latter profiles molecular lipid species distinct from total cholesterol/HDL-C/LDL-C. 2. Studies [47,48] used chest radiography scoring systems (DRHDS and DRED, respectively); information on radiograph readers (number and inter-rater reliability) was not reported in the original publications.

**Table 4:** Metabolic disorders and clinical outcomes in PTB: clinical, radiological, and pharmacological evidence.

Study (Ref)	Design	Population	Exposure	Timing	TB Stage	Outcome	Effect Estimate	Confounders/ Adjustment
Part 1. Glycemic/insulin-resistance indices and radiological severity								
Chen et al., 2022 [64]	Retrospective cross-sectional	247 T2DM-PTB (China); ATT ≤ 1 week	TyG index = Ln[TG × FBG/2]; Q1: 6.85–8.66, Q4: 9.66–10.54; per 1-unit	Baseline (pre-/early ATT)	Active T2DM-PTB	CT: air bronchial sign, multiple cavities, thick-walled cavity	Q4 vs. Q1: air bronchial aOR 8.07 (1.702–44.026); multiple-cavities aOR 7.75 (1.714–31.95); thick-walled aOR 7.82 (1.865–34.706). Per 1-unit: multiple-cavities aOR 4.10 (1.26–13.31)	Age, sex, BMI, BP, smoking, alcohol, dyslipidemia, comorbidities, FBG, HbA1c, antidiabetic meds, lipids, coagulation; Bonferroni
Barreda et al., 2020 [65]	Retrospective cross-sectional	132 PTB (73 normo, 41 prediabetes, 18 diabetes; Peru)	Dysglycemia (ADA: FPG/HbA1c/OGTT)	Baseline/pre-ATT	Active PTB	Radiographic diversity (≥3 lesion types, ≥4 lesions)	Hyperglycemia vs. normo: 3 types 59% vs. 26% (*p* < 0.001). Adj.: prediabetes→3 types *p* = 0.003, ≥4 lesions *p* = 0.001; diabetes → 3 types *p* < 0.01, ≥4 lesions *p* = 0.01. No OR CIs reported	Age, sex, prior TB, hemoglobin, AFB smear ≥2+
Part 2. TB-DM pathobiology								
Brake et al., 2025 [66]	Prospective cohort (TANDEM)	TB-DM, TB, HC	Olink inflammation + NMR lipidomics (VLDL, ApoB, sphingolipids)	Baseline + month 2 ATT	Active PTB	Radiographic scores; atherogenic/inflammatory profile	TB-DM: sustained ↑VLDL, ApoB, inflammatory mediators vs. TB-only/HC; correlated with severity	Age, sex, BMI, DM status, ATT duration
Dong et al., 2018 [67]	Matched case–control	DM-TB vs. NDM-TB pairs	Low-HDL-C, low-LDL-C phenotypes (SN/SP)	Baseline	Active PTB	Necrosis, fibro-encapsulation, cavitation	Low-LDL-C aOR 0.421; low HDL-C trend aOR 0.165 (*p* = 0.054). SN → caseous; SP → thick-walled cavities	Matched on clinical variables
Part 3. Comorbidity, safety, and pharmaco-modification								
Lim et al., 2023 [68]	Prospective cohort (COSMOTB)	PTB with metabolic disorders	Metabolic disorders (IR, HTN, obesity, dyslipidemia)	Baseline/during ATT	Active PTB	DILI	Metabolic disorders→DILI: aHR 2.85 (1.01–8.07)	Age, sex, DM, BMI, ATT regimen
Degner et al., 2018 [69]	Retrospective cohort	2416 DS-PTB (699 DM); metformin sub: 634 DM (Taiwan)	Metformin (ITT ≤ 30 d; high-adherence ≥ 80%)	2000–2013	Active PTB-DM	All-cause mortality; culture conversion	DM vs. non-DM: aOR 1.91 (1.51–2.40). Metformin ITT: aHR 0.56 (0.39–0.82); ≥80%: aHR 0.41 (0.21–0.78)	Age, sex, CKD, cancer, cavitary, adherence; CKD 3b + excluded
Meng et al., 2024 [70]	Nested case–control (IATB China)	2047 PTB-DM; 563 unfavorable/1484 favorable	HbA1c < 8% (good) vs. ≥8% (poor); statin use ≥7 d	Pre-ATT/during ATT	Active PTB-DM	Unfavorable outcome (death/failure/LTFU/ongoing)	Good-control OR 0.404 (0.317–0.542); statin OR 0.553 (0.412–0.712); good control+statin OR 0.394 (0.264–0.521). RERI 0.388, AP 0.914, SI 0.786	Sex, age, smear, ACCI, DILI, regimen, CRP, globulin

Abbreviations: aHR, adjusted hazard ratio; aOR, adjusted odds ratio; ATT, anti-tuberculosis treatment; ↑, increase; DILI, drug-induced liver injury; DM, diabetes mellitus; DS-PTB, drug-susceptible PTB; IR, insulin resistance; T2DM, type 2 DM; TyG, triglyceride–glucose index; NDM-TB, non-diabetic tuberculosis. Notes: Cross-sectional studies (Part 1) do not imply causality. Ref. [64] restricted to T2DM-PTB. Ref. [65] reports *p*-values only (no OR CIs). Refs. [66,67] address pathobiology, not outcomes. Refs. [67,68,69,70] are observational; pharmaco findings cannot establish causality.

**Table 5:** Genetic (Mendelian randomization) and lipid-level evidence for metabolic–PTB associations.

Study (Ref)	Design	Population	Exposure	Timing	TB Stage	Outcome	Effect Estimate	Adjustment/ Instruments
Chidambaram et al., 2021 [51]	Retrospective cohort	514 DS-PTB with baseline lipids (NTUH Taiwan)	LDL/HDL/TC/TG tertiles (baseline ±1 month)	Baseline→9-month ATT	Active DS-PTB	9-month all-cause & infection-related mortality; CRP, WBC, NL ratio	HDL 3rd tertile: all-cause aHR 0.17 (0.07–0.44), infection-related aHR 0.13 (0.03–0.53). TC 3rd: all-cause aHR 0.30 (0.14–0.65), infection aHR 0.26 (0.09–0.72). LDL/TG NS after adj.	Age, sex, BMI, CCI, transplant, alcohol, smoking, smear, cavitary, metformin, statin, CCB; BMI no effect modification
Du et al., 2024 [69]	Two-sample & multivariable MR (IVW primary)	BBJ GWAS: 7800 PTB/170,871 controls (Japanese)	14 metabolic traits (genetic liability)	Lifetime	Incident active PTB	PTB occurrence	IVW MVMR: HDL-C OR 2.798 (1.484–5.274); LDL-C OR 4.027 (1.140–14.219); TG OR 2.548 (1.269–5.115); TC OR 0.131 (0.028–0.607). T2DM OR 1.219 (1.040–1.429)	IVs *p* < 5 × 10^−6^, LD clump, PhenoScanner pleiotropy

Abbreviations: aHR, adjusted hazard ratio; aOR, adjusted odds ratio; ATT, anti-tuberculosis treatment; BBJ, BioBank Japan; BMI, body mass index; CCB, calcium channel blocker; CCI, Charlson comorbidity index; CRP, C-reactive protein; DS-PTB, drug-susceptible pulmonary tuberculosis; GWAS, genome-wide association study; HDL, high-density lipoprotein; HDL-C, high-density lipoprotein cholesterol; HR, hazard ratio; IVs, instrumental variables; IVW, inverse-variance weighted; LD, linkage disequilibrium; LDL, low-density lipoprotein; LDL-C, low-density lipoprotein cholesterol; MR, Mendelian randomization; MVMR, multivariable Mendelian randomization; NL ratio, neutrophil–lymphocyte ratio; NS, not significant; NTUH, National Taiwan University Hospital; OR, odds ratio; PTB, pulmonary tuberculosis; T2DM, type 2 diabetes mellitus; TC, total cholesterol; TG, triglycerides; WBC, white blood cell count. Notes: Ref. [51] addresses lipid-level–mortality and systemic inflammation; LDL and TG were not independently associated with mortality after adjustment. Ref. [69] is a Mendelian randomization study, not an observational cohort; effect estimates are IVW odds ratios reflecting genetic-liability associations with incident active PTB and must not be directly compared with AOR/HR/OR from clinical studies in Table 4.

**Table 6:** Lipid levels and survival/inflammation in PTB: detailed tertile effects (supplementary to Table 5).

Study	Lipid	Tertile	All-Cause aHR (95% CI)	Infection-Related aHR (95% CI)	Inflammatory Marker Effect
Chidambaram 2021 [51]	HDL	3rd vs. 1st	0.17 (0.07–0.44)	0.13 (0.03–0.53)	↓CRP, WBC, NL ratio
Chidambaram 2021 [51]	TC	3rd vs. 1st	0.30 (0.14–0.65)	0.26 (0.09–0.72)	↓CRP, WBC, NL ratio
Chidambaram 2021 [51]	LDL	3rd vs. 1st	0.60 (0.24–1.49) NS	0.42 (0.11–1.67) NS	CRP only
Chidambaram 2021 [51]	TG	3rd vs. 1st	1.08 (0.65–1.81) NS	1.14 (0.58–2.24) NS	None

Abbreviations: aHR, adjusted hazard ratio; CI, confidence interval; ↓, decreased; CRP, C-reactive protein; HDL, high-density lipoprotein; LDL, low-density lipoprotein; NL ratio, neutrophil–lymphocyte ratio; NS, not significant; PTB, pulmonary tuberculosis; TC, total cholesterol; TG, triglycerides; WBC, white blood cell count. Note: This table presents tertile-specific effect estimates for the lipid–mortality analyses (Ref. [51]) summarized in Table 5.

**Table 7:** Mendelian randomization evidence: detailed genetic instruments and validation (supplementary to Table 5).

Study	Design Detail	Instruments	Pleiotropy/Sensitivity	Key Findings (Full)
Du et al., 2024 [69]	Two-sample IVW + MR-Egger + weighted median; MVMR	14 traits; IV *p* < 5 × 10^−6^; LD R^2^ < 0.01; clump 10,000 kb	PhenoScanner; MR-PRESSO; leave-one-out; Cochran’s Q	See Table 5 Part 1; full OR table available on request

Abbreviations: IVW, inverse-variance weighted; IV, instrumental variable; LD, linkage disequilibrium; MR, Mendelian randomization; MR-PRESSO, Mendelian Randomization Pleiotropy RESidual Sum and Outlier; MVMR, multivariable Mendelian randomization; OR, odds ratio. Note: This table provides detailed genetic instruments and sensitivity analyses for the Mendelian randomization study (Ref. [69]) summarized in Table 5.

## Data Availability

The data used in this study are derived from the peer-reviewed literature cited in the reference list. No new data were collected, generated, or analyzed in this study. Therefore, data sharing is not applicable to this article.

## Abbreviations

The following abbreviations are used in this manuscript:

aOR	adjusted odds ratio
ACC1	acetyl-CoA carboxylase 1
ACACA	acetyl-CoA carboxylase alpha
AGEs	advanced glycation end products
ApoB	apolipoprotein B
APCs	antigen-presenting cells
ASM	acid sphingomyelinase
ATT	anti-tuberculosis treatment
ATP	adenosine triphosphate
AUC	area under the curve
CD1	cluster of differentiation 1
CE	cholesteryl esters
Cer	ceramide
CI	confidence interval
CXCR5	C-X-C chemokine receptor type 5
DILI	drug-induced liver injury
DM	diabetes mellitus
DN1Tg	double-negative 1 T cell receptor transgenic
DR-TB	drug-resistant tuberculosis
DS-PTB	drug-sensitive pulmonary tuberculosis
FAO	fatty acid oxidation
HbA1c	glycated hemoglobin A1c
HDL-C	high-density lipoprotein cholesterol
HDTs	host-directed therapies
HR	hazard ratio
IFN-γ	interferon gamma
IL-1β	interleukin-1β
IL-2	interleukin-2
IL-17A	interleukin-17A
IL-18	interleukin-18
IPT	isoniazid preventive therapy
LDL-C	low-density lipoprotein cholesterol
LTBI	latent tuberculosis infection
LPS	lipopolysaccharide
MA	mycolic acid
MDR-TB	multi-drug-resistant TB
MHC	major histocompatibility complex
Mtb	Mycobacterium tuberculosis
NCEP ATP III	National Cholesterol Education Program Adult Treatment Panel III
NFAT	nuclear factor of activated T cells
NF-κB	nuclear factor kappa B
NLRP3	nucleotide-binding oligomerization domain-like receptor family, pyrin domain-containing 3
NTUH	National Taiwan University Hospital
OGTT	oral glucose tolerance test
OR	odds ratio
PD-1	programmed cell death protein-1
PD-L1	programmed death-ligand 1
PDCD1	programmed cell death 1 gene
PPARγ	peroxisome proliferator-activated receptor γ
PTB	pulmonary tuberculosis
RAGE	receptor for advanced glycation end products
RCTs	randomized controlled trials
RILP	Rab-interacting lysosomal protein
ROS	reactive oxygen species
RORγt	retinoic acid receptor-related orphan receptor γt
T2DM	type 2 diabetes mellitus
TACO	tryptophan-aspartate-containing coiled-coil protein
TCR	T cell receptor
Tcm	central memory T cell
TB	Tuberculosis
TB-DM	tuberculosis–diabetes mellitus
TC	total cholesterol
TG	triglycerides
Th1	T helper 1
TLR2	Toll-like receptor 2
TNF-α	tumor necrosis factor alpha
TOX	thymocyte selection-associated high mobility group box protein
TXNIP	thioredoxin-interacting protein
TyG	triglyceride–glucose
VLDL	very low-density lipoprotein
WHO	World Health Organization
